# Switchable Multifunctional Terahertz Metamaterials Based on the Phase-Transition Properties of Vanadium Dioxide

**DOI:** 10.3390/mi13071013

**Published:** 2022-06-27

**Authors:** Zhanshuo Sun, Xin Wang, Junlin Wang, Hao Li, Yuhang Lu, Yu Zhang

**Affiliations:** College of Electronic Information Engineering, Inner Mongolia University, Hohhot 010021, China; 32056106@mail.imu.edu.cn (Z.S.); 32056056@mail.imu.edu.cn (H.L.); 32156012@mail.imu.edu.cn (Y.L.); 32056139@mail.imu.edu.cn (Y.Z.)

**Keywords:** terahertz metamaterials, vanadium dioxide, electromagnetically induced transparency-like, polarization converter

## Abstract

Currently, terahertz metamaterials are studied in many fields, but it is a major challenge for a metamaterial structure to perform multiple functions. This paper proposes and studies a switchable multifunctional multilayer terahertz metamaterial. Using the phase-transition properties of vanadium dioxide (VO_2_), metamaterials can be controlled to switch transmission and reflection. Transmissive metamaterials can produce an electromagnetically induced transparency-like (EIT-like) effect that can be turned on or off according to different polarization angles. The reflective metamaterial is divided into I-side and II-side by the middle continuous VO_2_ layer. The I-side metamaterials can realize linear-to-circular polarization conversion from 0.444 to 0.751 THz when the incident angle of the y-polarized wave is less than 30°. The II-side metamaterials can realize linear-to-linear polarization conversion from 0.668 to 0.942 THz when the incident angle of the y-polarized wave is less than 25°. Various functions can be switched freely by changing the conductivity of VO_2_ and the incident surface. This enables metamaterials to be used as highly sensitive sensors, optical switches, and polarization converters, which provides a new strategy for the design of composite functional metamaterials.

## 1. Introduction

The electromagnetically induced transparency (EIT) effect has attracted extensive attention since it was proposed and discovered by Harris et al. [1,2]. It is a destructive quantum interference between two different excitation transition pathways in atomic systems. Due to the suppression of the electronic transition, an obvious transmission window is generated at the frequency of the original absorption resonance [3]. The EIT window has a large group refractive index, so it is suitable for optical switches and slow-light devices [4,5]. The realization of EIT in atomic systems requires a high-intensity pulsed laser and extremely low experimental temperature, which affect the application and development of EIT technology. In 2008, Zhang et al. [6] used metamaterials to achieve electromagnetically induced transparency-like (EIT-like) effects at room temperature, which not only avoided the various constraints of EIT in atomic systems but also increased the controllability of the EIT. EIT-like effects can be achieved by introducing bright mode and dark mode with different resonance frequencies and intensities into the metamaterials and controlling the near-field coupling between them. Currently, EIT-like metamaterials (EIT-like MM) are widely used in many fields, such as high-sensitivity sensors [7,8,9], optical switches [10,11,12], slow-light devices [13,14], and modulators [15,16].

The effective regulation of electromagnetic wave polarization is of great significance in the fields of electromagnetic wave communication, terahertz (THz) imaging, and fundamental physics [17,18,19]. As polarization-control elements of electromagnetic waves, traditional polarization converters mainly rely on the phase accumulation on the propagation path of electromagnetic waves to achieve polarization regulation, which has the characteristics of large size and difficult integration [20,21]. In 2007, Hao et al. [22] proposed a reflective metamaterial for polarization conversion. Due to the advantages of thin thickness, low loss, and easy integration, metamaterial polarization converters quickly caught the attention of researchers [23,24]. In 2013, Grady et al. [25] proposed a metamaterial polarization converter suitable for the THz frequency band, which greatly expanded the application frequency range of metamaterial polarization converters.

Metamaterials with two-dimensional materials or phase-change materials can perform the dynamic switching of functions; breaking through the application limitations of single-function metamaterials, they are rapidly becoming an area of research focus in the field of metamaterials. Wen et al. [26] designed metamaterials with vanadium dioxide (VO_2_) resonant surfaces, and by changing the conductivity of the VO_2_, the transmittance can be adjusted and the opening and closing of the resonant peaks can be achieved. Yao et al. [27] proposed a metamaterial with a graphene resonant surface; by changing the Fermi level of the graphene, the polarization conversion range can be adjusted and a high polarization conversion rate can be maintained in specific frequency ranges. Li et al. [28] designed a multifunctional metamaterial based on Ge_2_Sb_2_Te_5_ (GST). Switching between transmissive quarter-wave plates (QWP) and half-wave plates (HWP) can be achieved by controlling the crystalline state of the GST. Song et al. [29] designed a multilayer THz metamaterial containing square VO_2_ layers and rectangular metal layers. The switching of the broadband absorber and the broadband linear polarization converter can be achieved by tuning the insulating and conducting states of the VO_2_. Li et al. [30] proposed a multilayer metamaterial containing a resonant layer of VO_2_ and metal combinations, a continuous VO_2_ layer, and a continuous graphene layer. When VO_2_ is in the conducting state, metamaterials can achieve broadband absorption in the 0.4 THz bandwidth. When the VO_2_ is in the insulating state, the switching of dual-frequency EIT-like and broadband EIT-like is achieved by changing the Fermi level of the graphene. Liu et al. [31] achieved switching between dual-frequency EIT-like and linear-to-circular polarization conversion using an intermediate continuous VO_2_ layer. These metamaterials have many functions, which increases their application value. 

In this paper, the development of a multifunctional THz metamaterial is presented based on the phase transition properties of VO_2_. When the VO_2_ is in the insulating state, the electromagnetic wave incident on the I-side excites two bright modes, which are coupled with each other to produce EIT-like. The resonance mechanism of the EIT-like MM was analyzed in detail by analyzing the electric field distribution. The effects of the polarization angle and structural parameters on the EIT-like were also studied, and the appearance and disappearance of the EIT-like was controlled by adjusting the polarization angle. When the VO_2_ is in the conducting state, the metamaterials on both sides can be used as reflective polarization converters. The I-side metamaterials can achieve conversion from linear polarization to circular polarization in the range of 0.444 to 0.751 THz. The II-side metamaterials can achieve conversion from linear polarization to linear polarization in the range of 0.668 to 0.942 THz. The resonance mechanisms of the two polarization converters were analyzed in detail by using the surface current in a u–v coordinate system. The effects of the structural parameters and the incident angle of the polarization wave on the polarization converter were also studied. The I-side metamaterials can be used as a QWP in the frequency range of 0.444–0.751 THz when the incident angle is less than 30°. The II-side metamaterials can be used as HWP in the frequency range of 0.668–0.942 THz when the incident angle is less than 25°. The proposed multilayer THz metamaterial shows potential for the active manipulation of the EIT-like effect and polarization conversion, and provides a new strategy for the design of multifunctional metamaterials.

## 2. Structure Model

**Figure 1:**
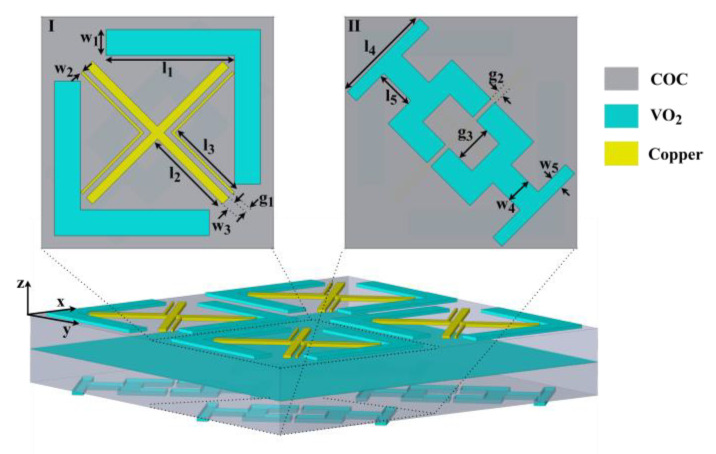
Unit structure and 2 × 2 periodic structure of metamaterials.

Figure 1 shows the structure of the multifunctional THz metamaterials, which is divided into two parts, the I-side and the II-side, by a VO_2_ layer with a thickness of 0.5 μm. For the I-side part, the 3 micrometer-thick surface resonator layer contains three differently shaped structures: X-shape (XS), small double-L-shape (SDLS), and large double-L-shape (LDLS). XS and SDLS are made of copper with electric conductivity of σ = 5.8 × 10^7^ S/m, and the material of LDLS is VO_2_. For the II-side part, surface resonator layer is composed of the VO_2_ resonant structure with a thickness of 4 μm. The intermediate insulating layer is composed of the cyclic olefin copolymer (COC) with a relative permittivity of 2.1 + 0.006i and a thickness of 30 μm. The structural parameters of metamaterial are as follows: $l_{1}$ = 100 µm, $l_{2}$ = 71.5 µm, $l_{3}$ = 65.5 µm, $l_{4}$ = 80 µm, $l_{5}$ = 30 µm, $g_{1}$ = 4.5 µm, $g_{2}$ = 4 µm, $g_{3}$ = 30 µm, $w_{1}$ = 20 µm, $w_{2}$ = 2 µm, $w_{3}$ = 7 µm, $w_{4}$ = 20 µm, $w_{5}$ = 10 µm. The metamaterial’s structure was simulated using the 3D full-wave electromagnetic field simulation software CST Studio Suite 2020. To simulate infinite periodic arrays, the periodic boundary conditions were set in the X and Y directions and the open boundary conditions were set in the Z direction.

VO_2_ is a temperature-dependent phase-change material whose electrical conductivity and relative permittivity change during the phase-transition process. In the THz frequency band, the relative permittivity of VO_2_ is described by the Drude model [32,33]:

(1) $$\varepsilon \left(\omega \right)=\varepsilon_{\infty }-\frac{\omega_{p}^{2}\left(\sigma \right)}{\omega^{2}+i\gamma \omega }$$

where $\varepsilon_{\infty }$ is the permittivity at infinite frequency, with a value of 12, and γ is the collision frequency, with a value of 5.75 × 10^13^ rad/s. The relationship between the plasma frequency $\omega_{p}\left(\sigma \right)$ and the conductivity σ is as follows:

(2) $$\;\;\omega_{p}^{2}\left(\sigma \right)=\frac{\sigma }{\sigma_{0}}\omega_{p}^{2}\left(\sigma_{0}\right)$$

where $\sigma_{0}$ = 3 × 10^5^ S/m, $\omega_{p}^{2}\left(\sigma_{0}\right)\;$ = 1.4 × 10^15^ rad/s. In the simulation, σ = 30 S/m represents the insulating state of VO_2_ at room temperature and σ  = 90,000 S/m represents the conducting state of VO_2_ at 89 °C [34]. The transition between VO_2_ insulating state and conducting state can produce the transmission and reflection conversion of metamaterials.

## 3. Electromagnetically Induced Transparency-like Effect of Metamaterials

**Figure 2:**
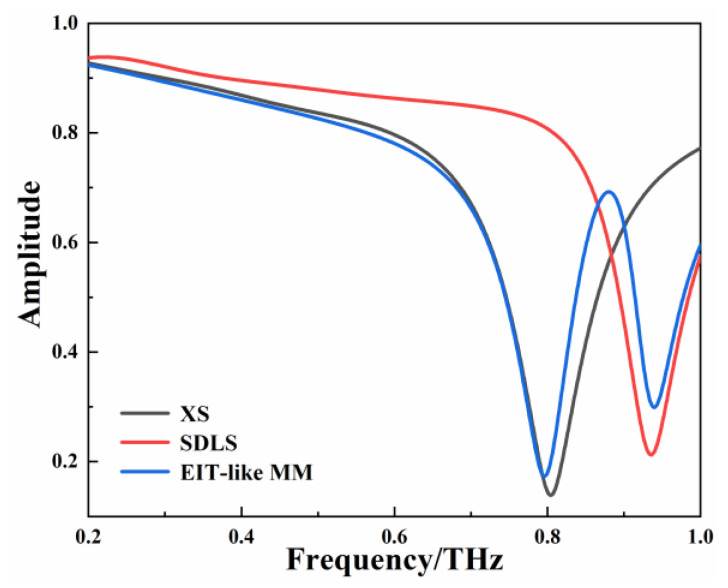
Transmission-amplitude curves of XS, SDLS, and EIT-like MM.

The metamaterial is excited by the incident THz wave to produce an EIT-like effect when the VO_2_ is in the insulating state. The metal resonant unit of the EIT-like MM is composed of XS and SDLS. The THz wave with the electric field along the *y*-axis and the wave vector along the *z*-axis is vertically incident on the surface of the metamaterial. Figure 2 shows the transmission curves of each individual XS, SDLS, and EIT-like MM resonator under the same polarization conditions. The XS and SDLS are excited by incident radiation and resonate at 0.805 THz and 0.935 THz, respectively. The resonant frequencies of the two bright modes are similar and the quality factors of the resonant windows are obviously different, which meets the necessary conditions for an EIT-like effect. The transmission curve of the EIT-like MM is coherently superimposed by the transmission curves of the bright modes. The EIT-like transmission window is formed between 0.796 THz and 0.939 THz, with a peak frequency of 0.881 THz and a transmission peak amplitude of 0.692.

**Figure 3:**
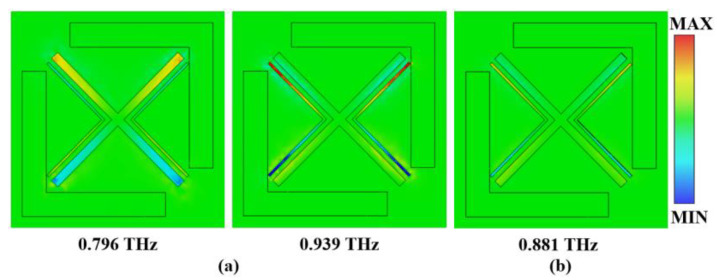
(**a**) Electric-field distribution of the trough frequencies; (**b**) electric-field distribution of the transmission-peak frequency.

The generation mechanism of the EIT-like was further analyzed based on the electric-field distribution of the metamaterial’s resonant surface. The electric-field distributions corresponding to the trough and peak frequencies are shown in Figure 3a,b. At the trough frequency of 0.796 THz, the bright modes are excited by the incident THz wave. The electric-field strengths of XS and SDLS are different. The electric field is mainly concentrated on the XS. The XS generates electric dipole resonance, and the weak coupling between the XS and the SDLS makes the electric dipole of the SDLS rotate under the action of the original electric field, so that its electric dipole moment turns to the direction of the external electric field. Therefore, the electric field on the SDLS is not concentrated at the ends of the L-shaped structure. At 0.939 THz, the SDLS produces electric dipole resonance and the electric field plays a leading role in the structure. The resonant intensities of the bright modes are different. Due to the weak hybridization between the bright modes, the electric-field strength of the structure is significantly weakened, and an obvious transmission window is induced at 0.881 THz.

**Figure 4:**
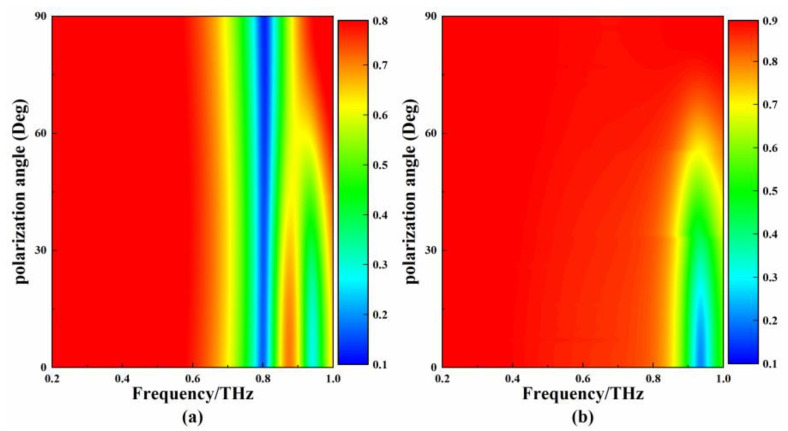
(**a**) The transmission curves of EIT-like MM corresponding to different polarization angles; (**b**) the transmission curves of SDLS corresponding to different polarization angles.

The EIT-like MM is a two-fold rotational symmetry structure, which is usually sensitive to changes in polarization angle [35]. To deeply study the effect of different polarization angles on the EIT-like characteristics, the transmission properties of the metamaterial under different polarization angles were simulated, and the results are shown in Figure 4a. The peak frequency and frequency range of the EIT-like window do not change significantly when the polarization angle is changed from 0° to 30°. As the polarization angle increases from 30° to 60°, the amplitude of the transmission peak and the frequency range of the transmission window decrease. When the polarization angle is greater than 60°, the EIT-like effect disappears and the metamaterial produces a single resonance. In summary, the appearance and disappearance of the EIT-like are related to the response of the bright modes to different polarization angles. The XS is a centrosymmetric structure and is insensitive to changes in polarization angle. Figure 4b shows the simulation results of the SDLS with different polarization angles. The degradation of the resonance corresponds to the degradation of the EIT-like, and the changes to the EIT-like are determined by the response of the SDLS to different polarization angles. The sensitivity of the metamaterial to the polarization angle can be applied in the field of optical switches.

**Figure 5:**
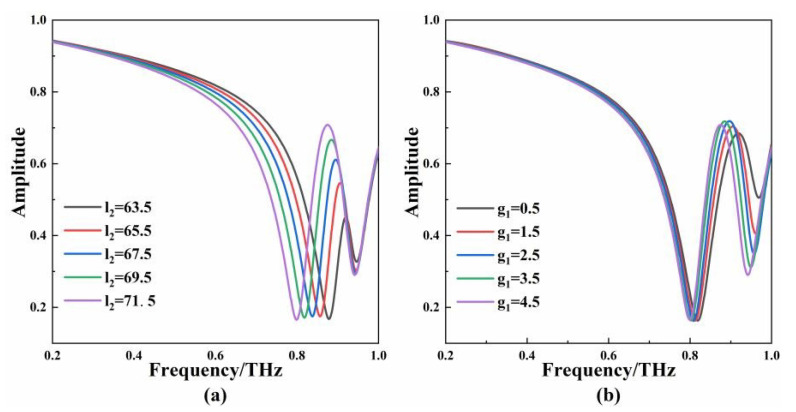
(**a**) The transmission curves corresponding to different lengths of $l_{2}$; (**b**) the transmission curves corresponding to different lengths of $g_{1}$.

In order to obtain further insights into the EIT-like characteristics, the effects of the structural parameters were simulated. Only one parameter was changed at a time, and the other parameters remained unchanged. Figure 5a shows that when the $l_{2}$ length increases, the transmission window is red-shifted, and the amplitude of the transmission peak becomes larger. The transmission peak of the EIT-like can be regulated by changing the length of $l_{2}$. Figure 5b shows the simulation results of changing $g_{1}$. When $g_{1}$ increases from 0.5 µm to 4.5 µm, the amplitude of the transmission peak remains unchanged, and the peak frequency is slightly red-shifted. It is worth noting that the full width at half maximum of the transmission window decreases significantly. The quality factor of the metamaterial sensor is defined as $Q=f_{EIT-like}/FWHM$. The $\;f_{EIT-like}$ is the peak frequency of the EIT-like window and FWHM is the full width at half maximum. When the metamaterial is used as the sensor, the increase in $g_{1}$ corresponds to a higher quality factor. 

## 4. Polarization Conversion Properties of Metamaterials

### 4.1. Simulation Results

At 89 °C, the continuous VO_2_ layer in the conducting state divides the metamaterials into I-side and II-side parts, which can be used as reflective polarization converters. To analyze the degree of the polarization conversion of the metamaterial when the y-polarized THz wave is incident, the Stokes parameters [36,37] are introduced as follows:

(3) $$\;S_{0}={\left|r_{yy}\right|}^{2}+{\left|r_{xy}\right|}^{2}$$

(4) $$\;\;\;S_{1}={\left|r_{yy}\right|}^{2}-{\left|r_{xy}\right|}^{2}$$

(5) $$S_{2}=2\left|r_{yy}\right|\left|r_{xy}\right|\cos\Delta \Phi$$

(6) $$S_{3}=2\left|r_{yy}\right|\left|r_{xy}\right|\sin\Delta \Phi$$

where $\left|r_{yy}\right|$ is the reflection coefficient of the y-to-y polarization conversion, $\left|r_{xy}\;\right|\;$ is the reflection coefficient of the y-to-x polarization conversion, and $\Phi_{yy}$ and $\Phi_{xy}$ are the phases corresponding to the reflected waves, $\Delta \Phi =\Phi_{yy}-\Phi_{xy}$. When $\left|r_{yy}\;\left|\;\approx \;\right|r_{xy}\right|$ and ∆Φ ≈ 2*n*π ± π/2 (*n* is an integer), the metamaterials can achieve linear-to-circular polarization conversion. The calculated ellipticity can be used to describe the performance of the polarization converter. The ellipticity, defined as $\chi =S_{3}/S_{0}$. χ = −1, indicates that the reflected THz wave is a typical right-hand circular polarization (RHCP) wave. By contrast, χ = 1 means that the reflected THz wave is a typical left-hand circular polarization (LHCP) wave. When $\left|r_{yy}\;\left|\;\approx \;\right|r_{xy}\right|$ and ∆Φ ≈ 2*n*π ± π (*n* is an integer), the metamaterial can realize linear-to-linear polarization conversion, and its polarization conversion characteristics can be described by the polarization conversion rate and the degree of linear polarization [38]. The polarization conversion rate is defined as $PCR={\left|r_{xy}\right|}^{2}/S_{0}$, and the degree of linear polarization is defined as $DoLP=\sqrt{S_{1}^{2}+S_{2}^{2}}/S_{0}$. PCR = 1 indicates that the incident y-polarized wave is completely transformed into the reflected x-polarized wave. DoLP = 1 means that the degree of linear polarization is the highest, and the reflected THz waves are stable linear polarization waves.

**Figure 6:**
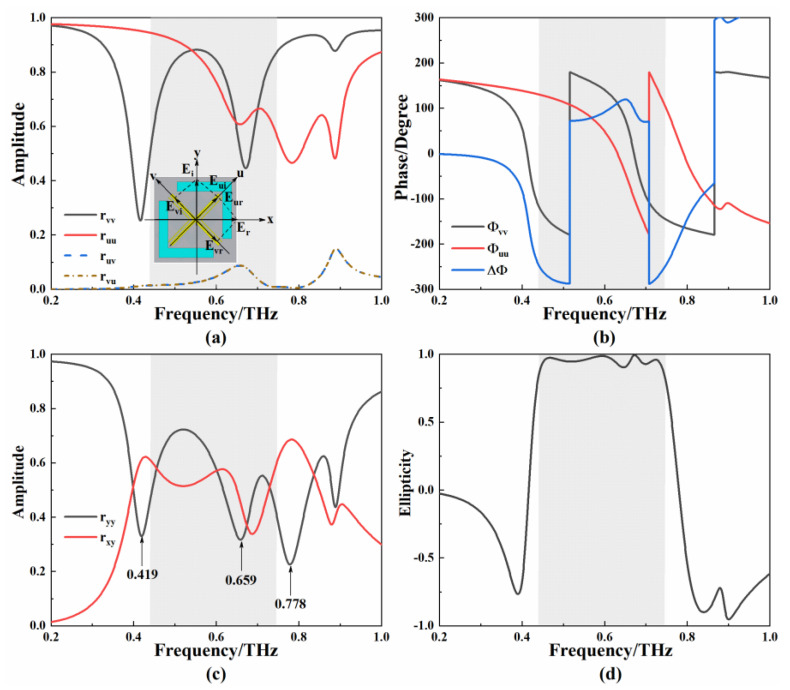
(**a**) Schematic diagram and simulation curve of the I-side metamaterial in u–v coordinate system; (**b**) phase difference of simulation curve of the I-side metamaterial in u–v coordinate system; (**c**) simulation curve of the I-side metamaterial in x–y coordinate system; (**d**) calculated ellipticity.

The u–v coordinate system is obtained by rotating the x–y coordinate system by 45° around the *z*-axis, where the incident and reflected waves are decomposed into mutually orthogonal u and v components [39,40]. The electric field of the incident wave is expressed as $E_{i}=E_{ui}u+E_{vi}v=\left|E_{ui}\right|exp\left(j\varphi \right)u+\left|E_{vi}\right|exp\left(j\varphi \right)v$. The electric field of the reflected wave is expressed as $E_{r}=E_{ur}u+E_{vr}v$. The symbols u and v are the unit direction vectors in the u-axis direction and v-axis direction, respectively. Jones calculus is used to explain the relationship between the incident and reflected waves [41]. The component of the reflected wave in the u-direction is expressed as $E_{ur}=\left|r_{uu}\right|exp\;\left(j\varphi_{uu}\right)E_{ui}+\left|r_{uv}\right|exp\;\left(j\varphi_{uv}\right)E_{vi}$. The component of the reflected wave in the V direction is expressed as $E_{vr}=\left|r_{vu}\right|exp\;\left(j\varphi_{vu}\right)E_{ui}+\left|r_{vv}\right|exp\;\left(j\varphi_{vv}\right)E_{vi}$. The symbols $r_{uu}$, $r_{vv}$, $r_{vu}$, and $r_{uv}$ represent the polarization of *u* to *u*, *v* to *v*, *u* to *v*, and *v* to *u*, respectively. In order to illustrate the polarization conversion properties of the metamaterial, the v- and u-polarized waves are incident from the I-side of the metamaterial, and the simulation results are shown in Figure 6a,b. In the frequency range of 0.444 to 0.751 THz, the cross-polarized reflection amplitudes are approximately equal to 0, the co-polarized reflection amplitudes are nearly equal, and the phase difference is close to 90° and −270°. The amplitude curves of $r_{yy}$ and $r_{xy}$ are shown in Figure 6c. The I-side metamaterial resonates at 0.419 THz and 0.778 THz, which causes $r_{yy}$ and $r_{xy}$ to intersect between the two frequencies. The resonance at 0.659 THz brings the amplitudes of the reflected polarization waves close to each other. The calculated χ is shown in Figure 6d. In the frequency range of 0.444–0.751 THz, χ > 0.92 indicates that the I-side metamaterials can convert the y-polarized incident wave to the LHCP wave.

**Figure 7:**
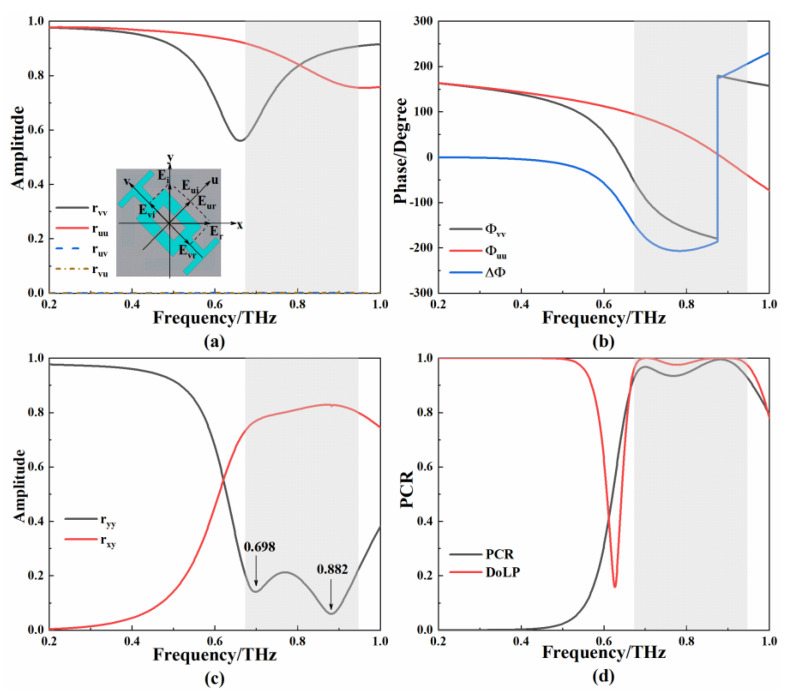
(**a**) Schematic diagram and simulation curve of the II-side metamaterial in u–v coordinate system; (**b**) phase difference of simulation curve of the II-side metamaterial in u–v coordinate system; (**c**) simulation curve of the II-side metamaterial in x–y coordinate system; (**d**) calculated PCR and DoLP.

Figure 7a,b show the simulation results of the II-side metamaterials in the u–v coordinate system. The amplitudes of $r_{vu}$ and $r_{uv}$ are 0. In the frequency range of 0.668–0.942 THz, the amplitudes of $r_{uu}$ and $r_{vv}$ are gradually close to unity, and the phase difference is close to ± 180°. The simulation and calculation results of the II-side metamaterials in the x–y coordinate system are shown in Figure 7c,d. The amplitude of $r_{xy}$ is higher than 0.72 and the amplitude of $r_{yy}$ is lower than 0.2 in the frequency range. The incident y-polarized wave is largely converted into an x-polarized wave. The PCR is greater than 0.94 and the DOLP is close to 1 in the frequency range, which indicates that the II-side metamaterial has the ability to achieve linear-to-linear polarization conversion.

### 4.2. Mechanism Analysis

**Figure 8:**
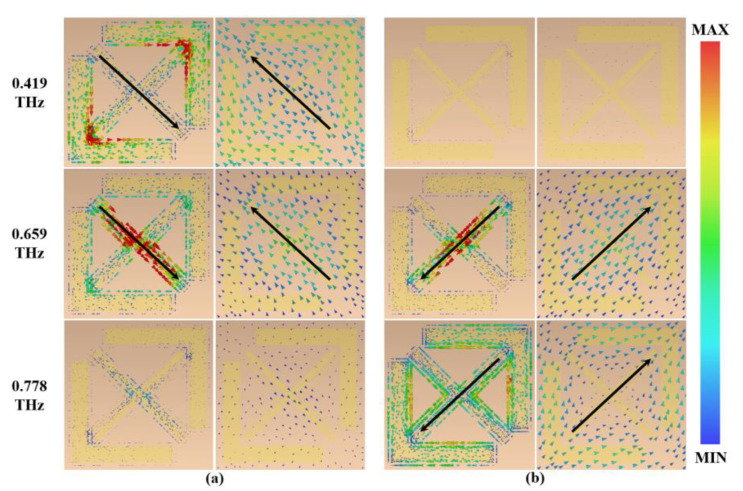
(**a**) Current distribution of the I-side metamaterial under v-polarization; (**b**) current distribution of the I-side metamaterial under u-polarization.

The mechanism of the linear-to-circular polarization conversion can be further analyzed by analyzing the resonance of $r_{yy}$ at 0.419 THz, 0.659 THz, and 0.778 THz. Figure 8a,b show the current distribution of the I-side metamaterials for v- and u-polarized incident waves, respectively. When the v-polarized wave is incident, the resonant layer currents at 0.419 THz and 0.659 THz are opposite to those of the VO_2_ layer, forming a loop to excite the magnetic dipole resonance. The resonant layer and VO_2_ layer have neither induced current nor resonance at 0.778 THz. Similarly, when the u-polarized wave is incident, the magnetic resonance is excited at 0.659 THz and 0.778 THz due to the opposite current directions of the metamaterial resonant layer and the VO_2_ layer. There is no resonance at 0.419 THz. The I-side metamaterial exhibits plasmonic resonance eigenmodes at 0.419 THz and 0.778 THz [42], where the eigenmodes are not a vector synthesis of the u and v components, but are generated by only one component. In the frequency range of 0.419–0.778 THz, the u and v components appear with the degradation of the eigenmodes. Magnetic resonance controls the magnitude and phase of the reflected electric field along the u- and v-axes to achieve the condition of circular polarization, and the superposition of the three resonances achieves broadband linear-to-circular polarization conversion.

**Figure 9:**
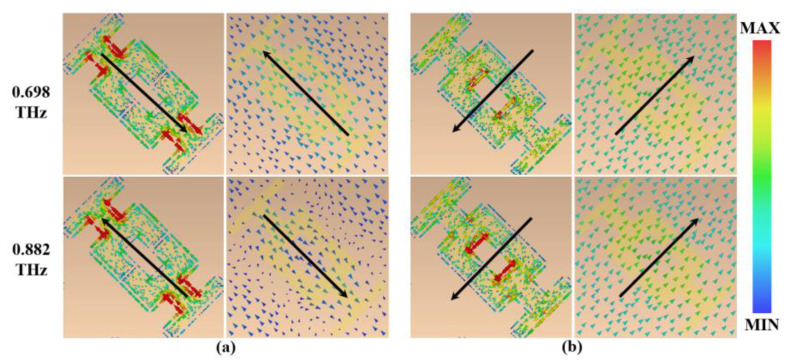
(**a**) Current distribution of the II-side metamaterials under v-polarization; (**b**) current distribution of the II-side metamaterials under u-polarization.

Similarly, the mechanism of linear-to-linear polarization conversion is explained by analyzing the resonance of $r_{yy}$ at 0.698 THz and 0.882 THz. Figure 9a,b show the current distribution of the II-side metamaterials for the v- and u-polarized incident waves, respectively. The II-side metamaterial induces opposite currents in the resonant layer and VO_2_ layer for the incident v-polarized waves and u-polarized waves. Therefore, the metamaterial produces magnetic resonances at 0.698 THz and 0.882 THz, which also show the degeneration and hybridization of plasmonic resonance eigenmodes [43]. Magnetic resonance controls the magnitude and phase of the reflected electric field along the u- and v-axes to achieve linearly polarized conditions, and, thus, linear-to-linear polarization conversion is achieved in the range of 0.668–0.942 THz.

### 4.3. Influence of Incidence Angle and Structural Parameters

**Figure 10:**
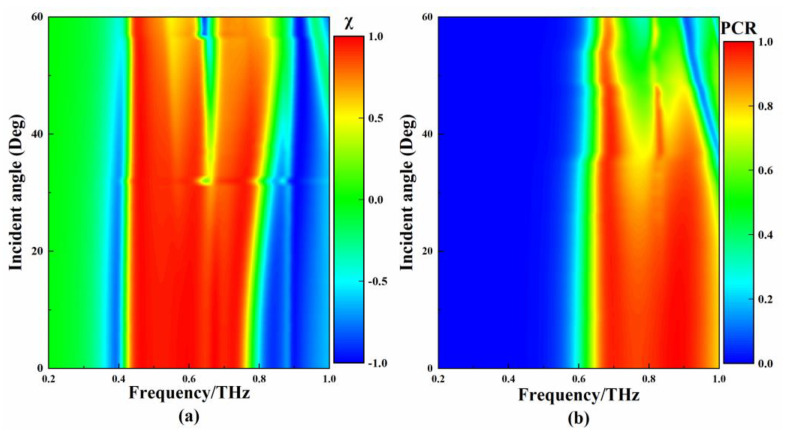
(**a**) Ellipticity corresponding to different incident angles of THz waves; (**b**) PCR corresponding to different incident angles of THz waves.

It is of great significance to investigate how the incidence angle (θ) of THz waves affect the polarization conversion properties of metamaterials. In order to understand the stability of the polarization converter to the change in θ, the θ of the THz waves was changed for simulation. Figure 10a,b show the simulation spectrum of the I-side and II-side metamaterials when changing the θ, respectively. For the I-side metamaterial, when the θ changes from 0° to 30°, the bandwidth of the polarization conversion increases slightly, and the ellipticity remains around 1. When the θ continues to increase, a dual-band polarization-converting metamaterial is achieved, but the ellipticity decreases significantly. For the II-side metamaterial, when the θ changes from 0° to 25°, the frequency range of the polarization conversion is stable, and the degree of polarization conversion remains high. However, when the θ continues to increase, the polarization conversion range gradually decreases. Based on the above research, when θ < 30°, the I-side metamaterial can be used as a stable QWP in the frequency range of 0.444–0.751 THz. When the θ < 25°, the II-side metamaterial can be used as a stable HWP in the frequency range of 0.668–0.942 THz.

**Figure 11:**
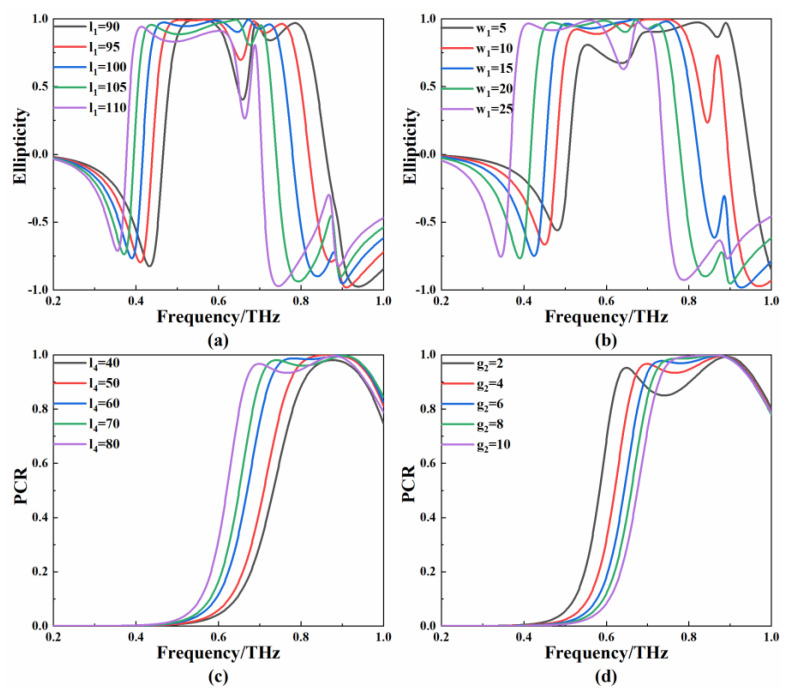
(**a**) Ellipticity corresponding to different lengths of $l_{1}$; (**b**) ellipticity corresponding to different lengths of $w_{1}$; (**c**) PCR corresponding to different lengths of $l_{4}$; (**d**) PCR corresponding to different lengths of $g_{2}$.

Except for the θ of the THz waves, the internal conditions, such as structural parameters, can also affect the polarization conversion properties. The influence of the key structural parameters on the polarization conversion properties of the metamaterial was studied. When the other parameters of the I-side metamaterial remain unchanged, the length of $l_{1}$ changes uniformly from 90 μm to 110 μm in steps of 5 μm; the simulation results of the ellipticity are shown in Figure 11a. Two narrow polarization conversion ranges are gradually merged into a broadband when $l_{1}$ increases from 90 μm to 100 μm. As $l_{1}$ continues to increase, the ellipticity decreases significantly at high frequencies. Figure 11b shows the calculated χ after changing the length of $w_{1}$. As $w_{1}$ increases from 5 µm to 25 µm, the bandwidth for χ > 0.9 first increases and then decreases. The $l_{4}$ and $g_{2}$ of the II-side metamaterial were changed separately for simulation, and the calculated PCR values are shown in Figure 11c,d. The increase in $l_{4}$ not only broadens the frequency range of the polarization conversion, but also maintains a high PCR in the range. As $g_{2}$ increases from 2 µm to 10 µm, the PCR grows closer to 1, although the corresponding bandwidth decreases.

## 5. Conclusions

In summary, this paper closely studied a multilayer terahertz metamaterial. The metamaterial exhibited different functions when the VO_2_ was in the insulating and conducting states. The metamaterial can exert an EIT-like effect that can be turned on or off, according to different polarization angles, when the VO_2_ is in the insulating state. The metamaterial was divided into I-side and II-side by the middle continuous VO_2_ layer when the VO_2_ was in the conducting state. The I-side metamaterial can achieve linear-to-circular polarization conversion from 0.444 to 0.751 THz when the incident angle of the y-polarized wave is less than 30°. The II-side metamaterial can achieve linear-to-linear polarization conversion from 0.668 to 0.942 THz when the incident angle of the y-polarized waves is less than 25°. The two types of polarization converter were freely switched by changing the incident surface of the y-polarized wave. Because the EIT-like window of the metamaterial is very sensitive to the change in the dielectric constant of the surrounding environment, the metamaterial can be used as a highly sensitive metamaterial sensor. The metamaterial can also be used as an optical switch according to the polarization-sensitive characteristics of the EIT-like. In a specific frequency range, the I-side and II-side metamaterials can be used as QWP and HWP, respectively. The multilayer terahertz metamaterial has potential applications in substance detection, ultrafast switches, THz communication, and THz imaging.
